# mir-150-5p inhibits the osteogenic differentiation of bone marrow-derived mesenchymal stem cells by targeting irisin to regulate the p38/MAPK signaling pathway

**DOI:** 10.1186/s13018-024-04671-6

**Published:** 2024-03-18

**Authors:** Jia-long Qi, Zhi-dong Zhang, Zhou Dong, Tao Shan, Zong-sheng Yin

**Affiliations:** 1 Department of Orthopedics, First Affiliated Hospital of Anhui Medical University, Hefei City, 230022 Anhui Province China; 2 Department of Spine Surgery, Hefei First People’s Hospital, Hefei City, 230061 Anhui Province China

**Keywords:** Osteoporosis, miR-150-5p, Irisin, Osteogenic differentiation, Bone marrow-derived mesenchymal stem cells

## Abstract

**Purpose:**

To study the effect of miR-150-5p on the osteogenic differentiation of bone marrow-derived mesenchymal stem cells (BMSCs), and further explore the relationship between its regulatory mechanism and irisin.

**Methods:**

We isolated mouse BMSCs, and induced osteogenic differentiation by osteogenic induction medium. Using qPCR to detect the expression of osteogenic differentiation-related genes, western blot to detect the expression of osteogenic differentiation-related proteins, and luciferase reporter system to verify that FNDC5 is the target of miR-150-5p. Irisin intraperitoneal injection to treat osteoporosis in mice constructed by subcutaneous injection of dexamethasone.

**Results:**

Up-regulation of miR-150-5p inhibited the proliferation of BMSCs, and decreased the content of osteocalcin, ALP activity, calcium deposition, the expression of osteogenic differentiation genes (Runx2, OSX, OCN, OPN, ALP and BMP2) and protein (BMP2, OCN, and Runx2). And down-regulation of miR-150-5p plays the opposite role of up-regulation of miR-150-5p on osteogenic differentiation of BMSCs. Results of luciferase reporter gene assay showed that FNDC5 gene was the target gene of miR-150-5p, and miR-150-5p inhibited the expression of FNDC5 in mouse BMSCs. The expression of osteogenic differentiation genes and protein, the content of osteocalcin, ALP activity and calcium deposition in BMSCs co-overexpressed by miR-150-5p and FNDC5 was significantly higher than that of miR-150-5p overexpressed alone. In addition, the overexpression of FNDC5 reversed the blocked of p38/MAPK pathway by the overexpression of miR-150-5p in BMSCs. Irisin, a protein encoded by FNDC5 gene, improved symptoms in osteoporosis mice through intraperitoneal injection, while the inhibitor of p38/MAPK pathway weakened this function of irisin.

**Conclusion:**

miR-150-5p inhibits the osteogenic differentiation of BMSCs by targeting irisin to regulate the/p38/MAPK signaling pathway, and miR-150-5p/irisin/p38 pathway is a potential target for treating osteoporosis.

**Supplementary Information:**

The online version contains supplementary material available at 10.1186/s13018-024-04671-6.

## Introduction

Osteoporosis (OP) is an age-related bone disease in which decreased bone density and bone strength are the main features of patients with OP [1]. Although the pathogenesis of OP is different due to different causes, the key to the treatment of osteoporosis is to promote bone formation and inhibit bone resorption to replace the lost bone tissue [2, 3]. Bone marrow mesenchymal stem cells (BMSCs) are progenitor cells that are capable of self-renewal and have the potential for multidirectional differentiation such as adipogenesis, osteogenesis, and chondrogenesis [4, 5]. Importantly, promoting osteogenic differentiation of BMSCs is currently a hot topic in the treatment of osteoporosis [6, 7].

MicroRNA (miRNA) is a small molecule (∼ 22 nucleotides) single-stranded noncoding RNA encoded by endogenous genes that has an important regulatory role in the cell [8]. Increasing data suggests that miRNAs play an important role in the treatment of orthopedic diseases such as tendon injuries [9], osteoarthritis [10] and osteoporosis [11]. MiRNAs can be involved in regulating the differentiation of BMSCs through different pathways thereby inducing bone reconstruction [5, 12]. Some genes that are abnormally expressed in BMSCs of patients with osteoporosis, such as miR-150-5p, have been discovered through RNA-seq technology. Geng Y, et al. compared BMSCs gene expression between healthy and elderly patients with osteoporosis and found that 63 non-coding RNAs and 415 mRNAs were abnormally expressed, including the hub gene miR-150-5p and its downstream gene FNDC5 [13]. However, the detailed mechanism of miR-150-5p on the osteogenic differentiation of BMSCs has not been further studied by Geng Y, et al., but miR-150-5p has been found to inhibit the osteogenic differentiation of ankylosing spondylitis fibroblasts [14] and adipose-derived stem cells [15]. At the same time, it is well known that the protein encoded by the FNDC5 gene (iris) has been found to regulate the differentiation of BMSCs [16, 17], including promoting the osteogenic differentiation of BMSCs [18]. Therefore, miR-150-5p may be involved in the regulation of osteogenic differentiation of BMSCs by regulating the expression of FNDC5.

To study the effect of miR-150-5p on the osteogenic differentiation of BMSCs, we firstly build the up-regulation or down-regulation of miR-150-5p BMSCs, and found that miR-150-5p inhibited the osteogenic differentiation of BMSCs. Furthermore, by overexpression of FNDC5 and the use of p38/MAPK inhibitors, we found that miR-150-5p inhibited the osteogenic differentiation of BMSCs by targeting FNDC5/irisin to regulate the p38/MAPK signaling pathway.

## Materials and methods

### Isolation, cultivation, and identification of BMSCs

Isolate the tibia and femur of the mouse (C57BL/6, male, 6 weeks), and rinse the bone cavity using sterile PBS buffer. This study was approved by the Ethics Committee of Hefei First People’s Hospital (2022(76)). Collect the bone cavity irrigation fluid, centrifuge to collect the pellet, add complete mouse bone marrow mesenchymal stem cells (BMSCs) medium (MUXMX-80,011, Cyagen) to resuspend the pellet for culturing at 37 ℃ with 5% CO_2_. We changed the medium every 3 days and remove the un-adhered cells until the cells are overgrown and begin passaging. Third generation cells were used to analyze cell surface markers of BMSCs through flow cytometry, including CD29 (ab193591, abcam, UK), CD90 (ab226, abcam), CD34 (ab187568, abcam), and CD45 (ab305209, abcam).

### miRNA transfection into BMSCs

Synthesized miR-NC, miR-150-5p-mimic (Mimic, 4,464,066, ThermoFisher) and miR-150-5p-inhibitor (Inhibitor, AM17000, ThermoFisher) were transferred into BMSCs according to the instructions of the Lipo3000 transfection reagent kit (L300075, ThermoFisher, USA).

### Cell proliferation assay

1 × 10^4^ BMSCs were seeded into 96-well plates and cultured for different time (1, 3, 7 days and 14days). And then we removed the cell culture medium, washed with PBS buffer two times, followed by adding 10 µL of CCK8 reagent (40203ES60, YEASEN) and 90 µL of cell culture medium. After 2 h of cultivation (37 ℃ and 5% CO_2_), we measured the OD value of each well at a wavelength of 450 nm.

### Determination of osteocalcin, ALP activity and calcium deposition

15 days after the BMSCs were induced by the Osteogenic Differentiation Induction Medium Kit (OIM, PD-003, Procell), BMSCs were collected to detect the content of osteocalcin, alkaline phosphatase (ALP) activity and calcium deposition using mouse OC/BGP (Osteocalcin) ELISA Kit (D721126, Sangon Biotech), a ALP detection kit (LM-E800, Shanghai Lianmai Bioengineering Co., Ltd), and a calcium assay kit (S1063S, Beyotime), respectively.

### Real-time quantitative polymerase chain reaction

Real-time quantitative polymerase chain reaction (RT-qPCR) was used to determine the expression of mRNA and miRNA. In brief, RNAiso reagent (D9108A, Takara) was used to extract the total RNA from BMSCs, and prepared the cDNA using a reverse transcription kit (RR407A, Takara). At last, RT-qPCR was done using a qPCR master mix kit (A6006A, Promega). Primers for RT-qPCR was showed in supplementary materials Table 1.

### Western blot

RIPA lysis buffer (HY-K1001, MCE) was used to lyse BMSCs to extract total cellular protein, and the indicated total protein was analyzed by SDS-PAGE gel, and then the various protein molecules that have been isolated are transferred to the polyvinylidene fluoride (PVDF) membrane. After being blocked by 5% BSA for 1 h at room temperature, the PVDF membrane is immersed in a solution containing the primary antibodies against Runx2 (1:1000, ab192256, abcam), BMP2 (1:2000, ab284387), Osteocalcin (OCN, 1:500, ab93876, abcam), FNDC5 (1:1000, ab174833), p38 (1:1500, 9212, cell signaling technology) and phospho-p38 (1:500, 4511, cell signaling technology). Next, the PVDF membrane is immersed in a solution containing the secondary antibodies, such as goat anti-mouse IgG (1:2000, 9116, cell signaling technology) or goat-anti-rabbit IgG (1:2000, 7074, cell signaling technology). Finally, proteins were visualized with ECL solution (WBKLS0100, Beijing Xinjingke Biotechnologies Co., Ltd, China), followed by densitometry analysis using Imag J 3.0 (IBM, USA) and β-actin was loading as control.

### Over-expression of FNDC5

The upstream and downstream sequences of human FNDC5 gene (NM_153756.2) were searched in GenBank, primers were synthesized, and EcoRI digestion sites were introduced into the upstream and downstream primers according to the cloning requirements (supplementary materials Table 1). And then we cloned the FNDC5 gene into the pLenti-EF1a-EGFP-P2A-Puro-CMV-MCS-3Flag lentiviral expression vector (TSPLA10157, Testobio). Finally, a lentiviral shuttle plasmid carrying the FNDC5 gene was co-transfected with a helper packaging plasmid to co-transfect 293T cells to prepare lentivirus. Lentivirus was used to infect BMSCs (MOI = 100) to over-expression of FNDC5.

### Osteoporosis mice and treatment

A total of 40 C57BL/6 mice (male, 6 weeks) were sued in our research, and they were randomly divided into 4 groups after 1 week of adaptive feeding, namely, Healthy group, OP group, Irisin group, and Irisin + SB group. During the first 4 weeks of the research, all mice except the Healthy group were injected subcutaneously with dexamethasone (40 mg/kg/day for 4 weeks) to construct an osteoporosis model. For the next 8 weeks, mice in each group were given different drug treatments. Mice in Healthy group and OP group were given equal amounts of saline, and mice in the Irisin group were given recombinant irisin (120 ng/kg/week for 8 weeks, SPR8039, MERCK) through tail vein injection, and mice in Irisin + SB group were given recombinant irisin (120 ng/kg/week for 8 weeks, SPR8039, MERCK) through tail vein injection and given SB203580 (5 mg/kg/day for 8 weeks, A8254, Apexbio).

### Detection of serum Ca2+, 25(OH)D, osteocalcin and DPD

After 8 weeks of treatment with irisin or SB203580, peripheral blood was collected from mice by extracting eyeballs and centrifuging (at room temperature, 1000 g, 10 min) to collect serum. And we determined the serum level of 25(OH)D using a 25-OH-vitaminD (total ELISA kit; MSE-800, Bio TNT), and determined the serum level of osteocalcin using a mouse osteocalcin ELISA kit (ab285236, abcam). At the same time, we also collected the urinary of mice to detect the level of deoxypyridinoline (DPD) with a mouse DPD ELISA kit (SFJ20421, ShangHai shifeng biological technology co., ltd), which was standardized the content of creatinine in urine. In addition, the automatic biochemistry analyzer is used to detect serum level of Ca2+ (BS280, mindray).

### Statistical analysis

Data in the present study was recorded in Excel software (Microsoft, USA), analyzed in SPSS 20.0 (IBM, USA) and visualized by Graphpad prism 9.0 (Graphpad prism, USA). Measurement data was expressed as mean ± standard deviation (SD) with 3 independent replicates or 5 mice. Difference between more than two groups was compared using one-way ANOVA and two-way ANOVA. *P* < 0.05 indicated a significant difference.

## Results

### Mir-150-5p inhibited the proliferation and osteogenic differentiation of BMSCs

**Fig. 1:**
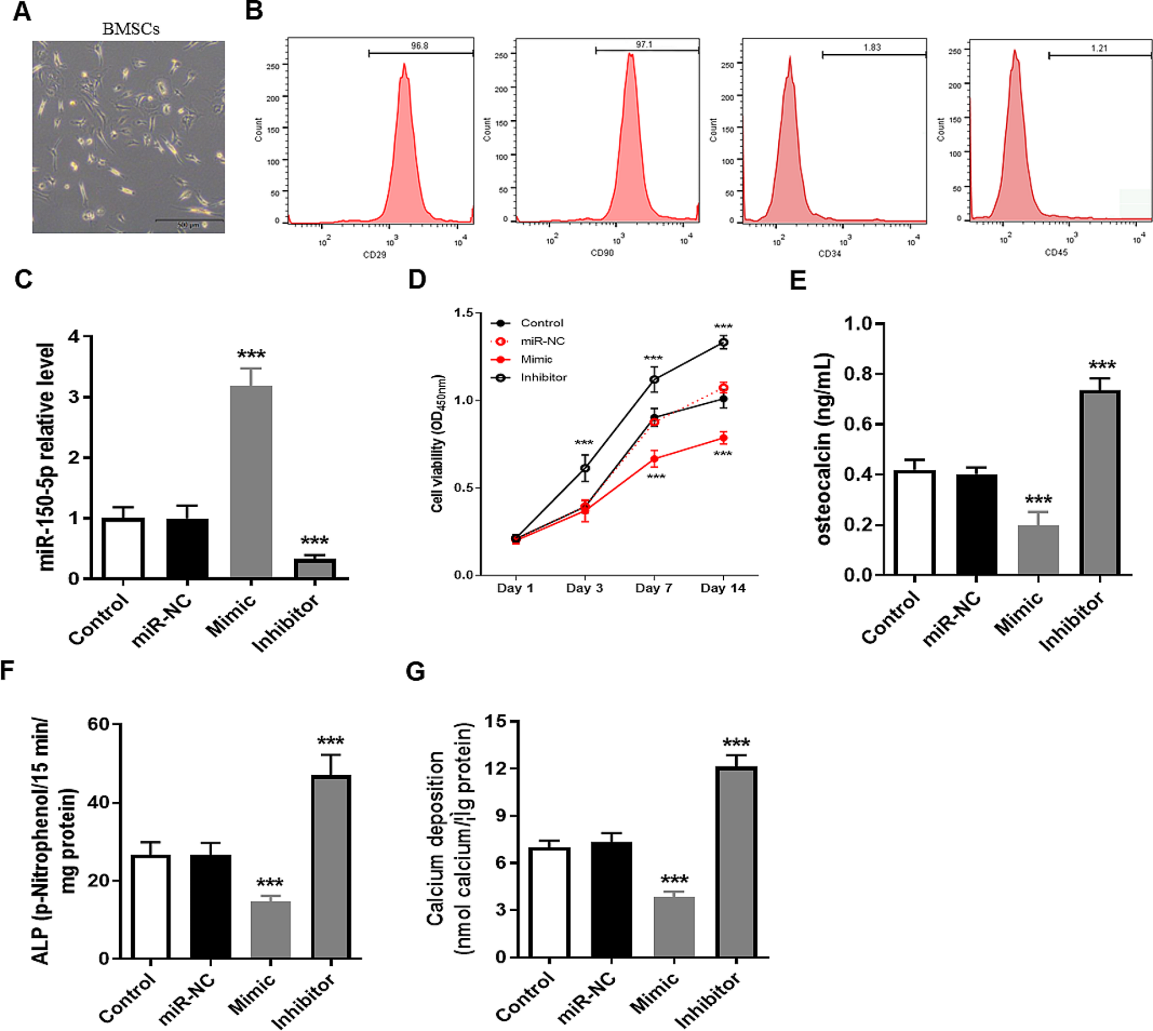
miR-150-5p inhibited the cell proliferation and osteogenic differentiation of mouse BMSCs. (**A**) Morphology of third-generation mouse BMSCs. (**B**) Identification of cell surface markers (CD29, CD34, CD45, CD90) in isolated mouse BMSCs using flow cytometry. (**C**) Detection of miR-150-5p expression levels in different cell lines of mouse BMSCs using RT-qPCR method. (**D**) Determination of cell viability in BMSCs transfected with Mimic or Inhibitor using CCK-8 assay kit. (**E-G**) After 15 days of osteogenic differentiation induction, osteocalcin content (**D**), ALP activity (**E**), and calcium deposition levels (**F**) were measured used a reagent kit. *** *P* < 0.001 vs. Control group using unpaired t test. miR-NC, transfection negative control. Mimic, stable overexpression of miR-150-5p. Inhibitor, stable knocking down miR-150-5p

BMSCs were isolated from the tibia and femur of mice for culture and the third passage was used for identification and follow-up studies (Fig. 1A). The result of flow cytometry found that a total of 96.8% cells positive for CD29, 97.1% cells positive for CD90, 1.83% cells positive for CD34 and 1.21% cells positive for CD45 (Fig. 1B). Therefore, isolated BMSCs could be used for further study. We then explored the effects of miR-150-5p on the proliferation and osteogenic differentiation of BMSCs through overexpression of miR-150-5p (Mimic) and knockdown of miR-150-5p (Inhibitor) (*P* < 0.001, Fig. 1C). The cell proliferation of BMSCs in the Inhibitor group was faster than that in the control group, while the cell proliferation of BMSCs in the Mimic group was slower than that in the control group (*P* < 0.001, Fig. 1D). Importantly, after 15 days of osteogenic differentiation induced by OIM medium, the content of osteocalcin (Fig. 1E), ALP activity (Fig. 1F) and calcium deposition (Fig. 1G) in the Mimic group were significantly lower than those in the control group, while those in the Inhibitor group were significantly higher than those in the control group (*P* < 0.001).

### Mir-150-5p inhibited the expression of osteogenic genes

**Fig. 2:**
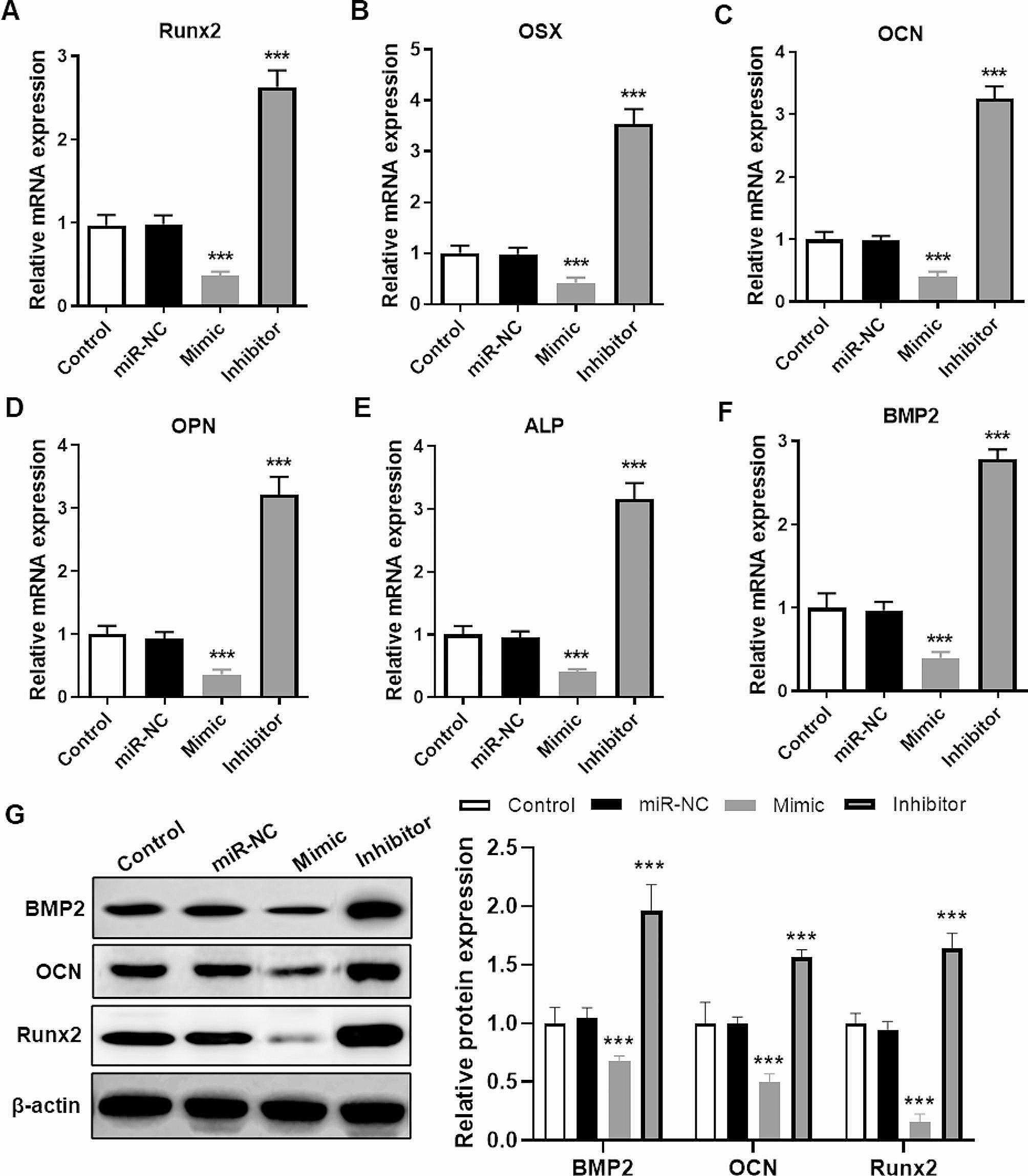
miR-150-5p inhibited osteogenic genes expression. (**A-F**) After 15 days of osteogenic differentiation induction, we determined the expression of Runx2 (A), OSX (B), OCN (C), OPN (D), ALP (E) and BMP2 (F) mRNA using RT-qPCR. (**G**) After 15 days of osteogenic differentiation induction, we determined the expression of BMP2, OCN and Runx2 protein using western blot. Statistical analysis of grayscale values of BMP2, OCN, and Runx2 protein bands. *** *P* < 0.001 vs. Control group using unpaired t test. miR-NC, transfection negative control. Mimic, stable overexpression of miR-150-5p. Inhibitor, stable knocking down miR-150-5p

After 15 days of osteogenic differentiation induced by OIM medium, we collected BMSCs to determine the expression of osteogenic genes, including Runx2, OSX, OCN, OPN, ALP and BMP2, and found that the mRNA expression of Runx2, OSX, OCN, OPN, ALP and BMP2 in the Mimic group were significantly lower than those in the control group, while those in the inhibitor group were significantly higher than those in the control group (Fig. 2A-F). Furthermore, the impact of miR-150-5p on the expression of the osteogenic proteins, including Runx2, BMP2, and OCN was assessed using western blot. The grayscale analysis of protein bands indicated that up-regulation of miR-150-5p significantly down-regulated the protein expression of Runx2, BMP2, and OCN, while down-regulation of miR-150-5p strongly up-regulated the expression of Runx2, BMP2, and OCN protein (Fig. 2G).

### Mir-150-5p targeted inhibits FNDC5 expression in mouse BMSCs

**Fig. 3:**
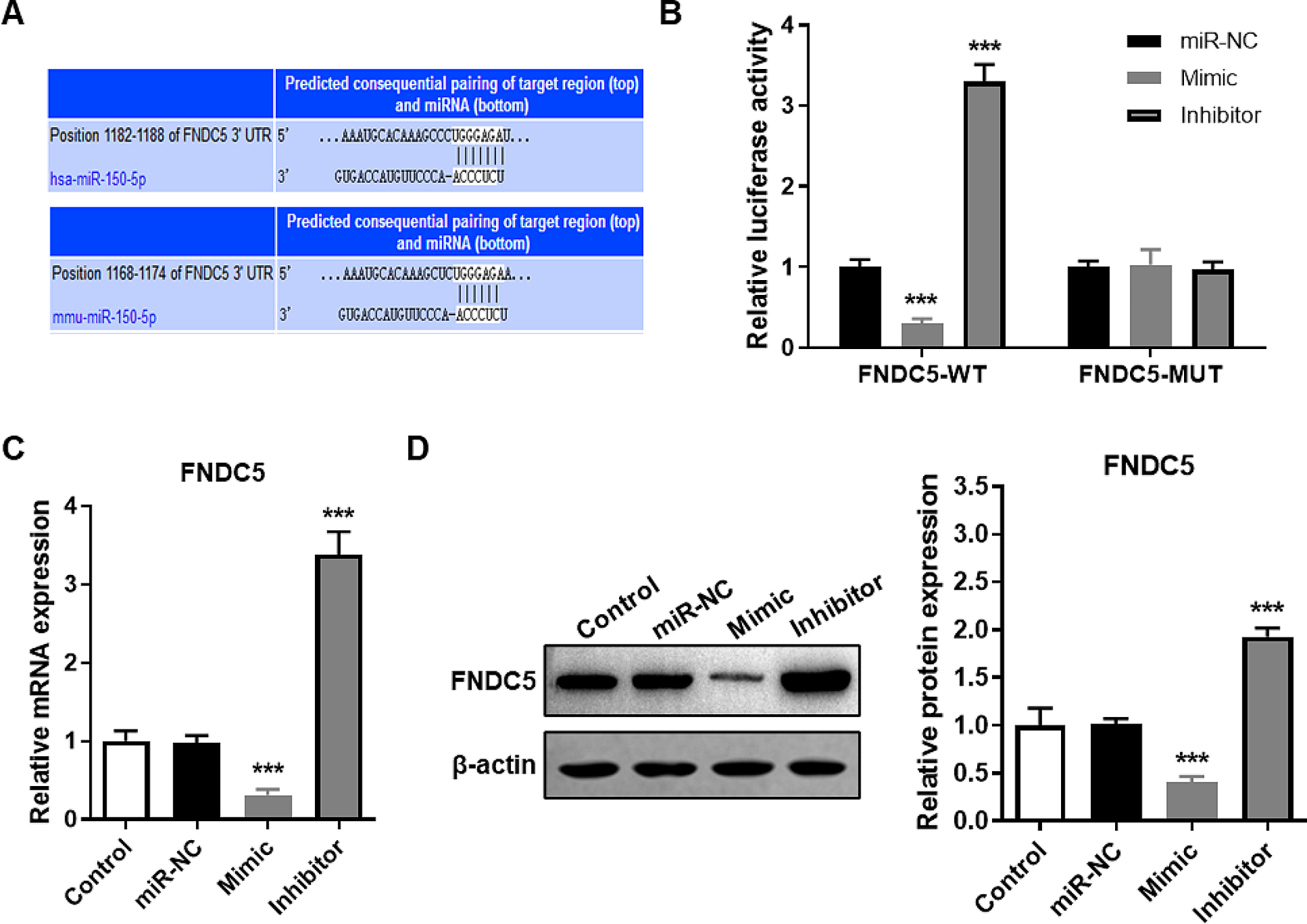
miR-150-5p targeted inhibits FNDC5 expression. (**A**) Predicted binding location of human (up) or mouse (down) miR-150-5p to the FNDC5 gene using bioinformatics in TargetScanHuman (https://www.targetscan.org/vert_71/). (**B**) Luciferase reporter containing WT or MUT FNDC5 sequences were transfected into mouse BMSCs prior to transfect with miR-150-5p mixed transfection reagent, and harvested the activity of luciferase. (**C-D**) Detection of FNDC5 mRNA (C) and protein (D) expression levels in mouse BMSCs with different transfection using RT-qPCR and western blot, respectively. *** *P* < 0.001 vs. Control group using unpaired t test. miR-NC, transfection negative control. Mimic, stable overexpression of miR-150-5p. Inhibitor, stable knocking down miR-150-5p

According to the public database (TargetScanHuman, https://www.targetscan.org/vert_71/) prediction of the target gene of miR-150-5p, we found that there is a complementary sequence between the FNDC5 gene and miR-150-5p (Fig. 3A). And the results of the luciferase gene reporter system showed that up-regulation or down-regulation of miR-150-5p did not affect the luciferase activity of mutant FNDC5 (MUT), but significantly changed the luciferase activity of wild type FNDC5 (WT) (Fig. 3B). Furthermore, the expression of FNDC5 mRNA (Fig. 3C) and protein (Fig. 3D) in BMSCs of the mimic group were remarkably lower than those in BMSCs of the control group, while those in BMSCs of the inhibitor group were strongly higher than those in BMSCs of control group. The results above indicated that miR-150-5p targeted inhibits FNDC5 expression.

### Mir-150-5p inhibited osteogenic differentiation of BMSCs by targeting FNDC5

**Fig. 4:**
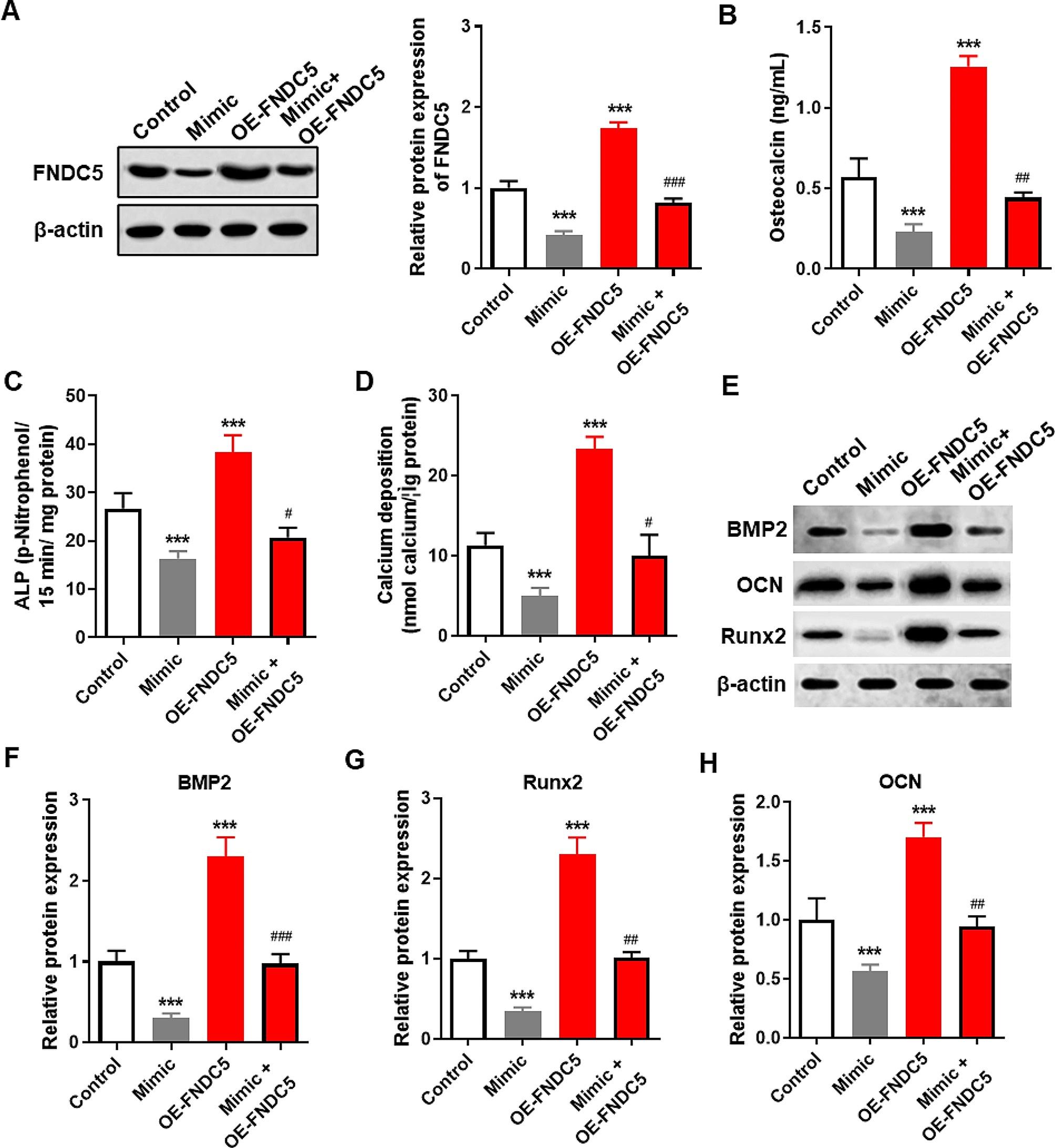
miR-150-5p inhibited osteogenic differentiation of BMSCs by targeting FNDC5. (**A**) Detection of FNDC5 protein expression levels in mouse BMSCs transfected with Mimic and/or OE-FNDC5 using western blot. (**B-D**) After 15 days of osteogenic differentiation induction in different cell lines of mouse BMSCs, we determined osteocalcin content (B), ALP activity (C), and calcium deposition levels (D). (**E-H**) Detection and statistical analysis of BMP2, OCN, and Runx2 protein levels in transfected BMSC by western blot. *** *P* < 0.001 vs. Control group using unpaired t test. # *P* < 0.05, ## *P* < 0.01 and ### *P* < 0.001 vs. Mimic group. Mimic, stable overexpression of miR-150-5p. OE-FNDC5, table overexpression of FNDC5. Mimic + OE-FNDC5, co-transfection of plasmid overexpressing FNDC5 and plasmid overexpressing miR-150-5p

To further investigate whether the effect of miR-150-5p on osteogenic differentiation of BMSCs was through the regulation of FNDC5 expression, we co-transfected BMSCs with Mimic and/or OE-FNDC5 to overexpress miR-150-5p, FNDC5 simultaneously and compared their differences in osteogenic differentiation. We also found that the expression of FNDC5 in BMSCs transfected with Mimic and OE-FNDC5 was strongly higher than that in BMSCs transfected with Mimic alone (Fig. 4A). The results of osteogenic differentiation showed that overexpression of miR-150-5p alone significantly decreased the content of osteocalcin, ALP activity and calcium deposition, while FNDC5 overexpression alone strongly increased the content of osteocalcin, ALP activity and calcium deposition (*P* < 0.001). Importantly, the content of osteocalcin, ALP activity and calcium deposition in BMSCs co-overexpressed miR-150-5p and FNDC5 was significantly higher than that of miR-150-5p overexpressed alone (*P* < 0.05, Fig. 4B-D). Similarly, overexpression of miR-150-5p alone significantly decreased the protein expression of BMP2, OCN and Runx2, while overexpression of FNDC5 alone strongly increased the expression of BMP2, OCN and Runx2 proteins (*P* < 0.001). Meanwhile, the expression level of BMP2, OCN and Runx2 in BMSCs co-overexpressed by miR-150-5p and FNDC5 was significantly higher than that of miR-150-5p overexpressed alone (*P* < 0.01, Fig. 4E-H). FNDC5 upregulation promoted osteogenic differentiation of BMSCs which is inhibited by overexpression of miR-150-5p.

### Mir-150-5p blocked p38-MAPK pathway by inhibiting FNDC5

**Fig. 5:**
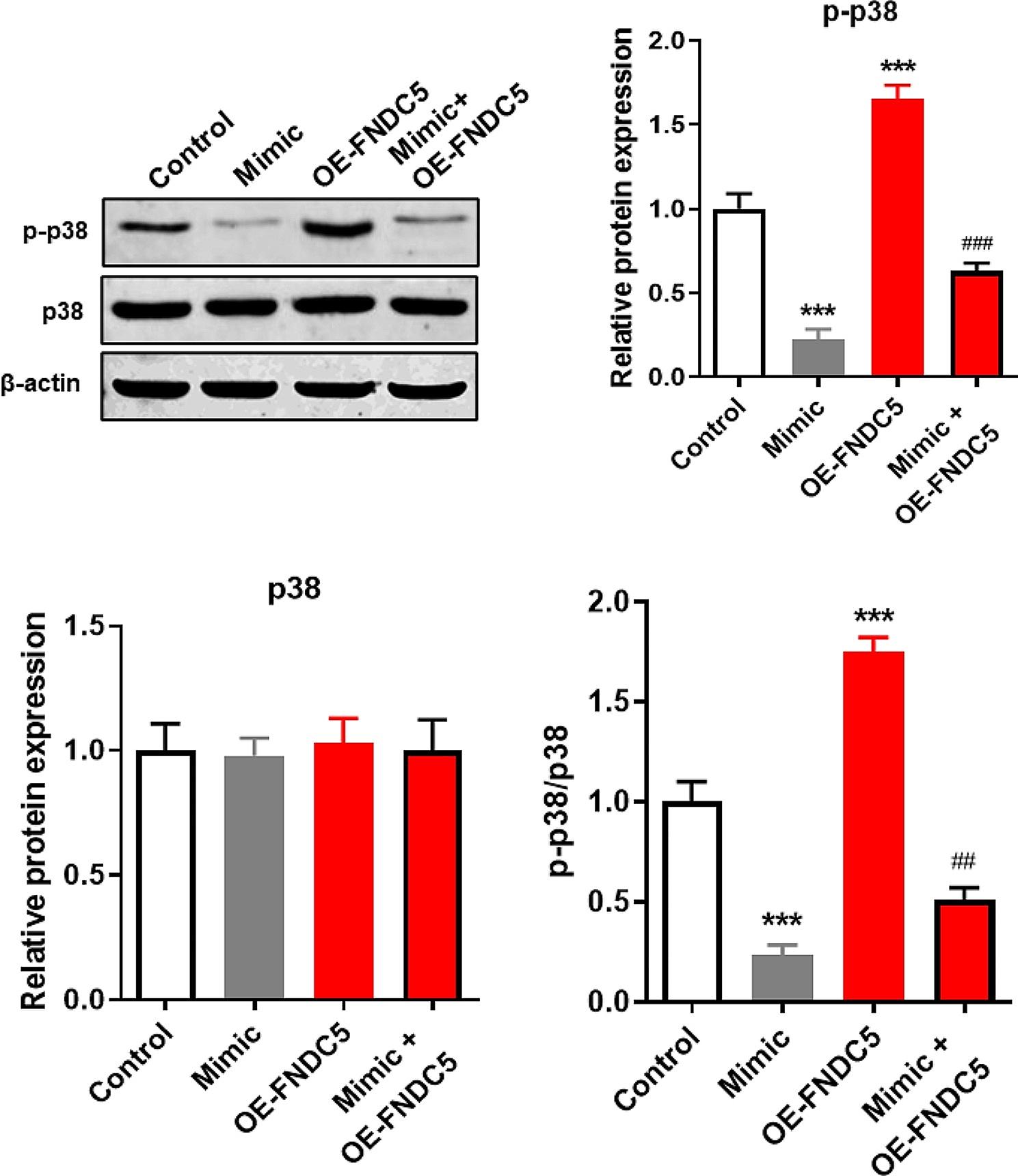
miR-150-5p blocked the p38-MAPK pathway by inhibiting FNDC5. After 15 days of osteogenic differentiation induction of mouse BMSCs transfected with different transfection, the expression of p38 and p-p38 protein was assessed by western blot and the relative grayscale values of p38, p-p38, and p-p38/p38 ratio were measured. *** *P* < 0.001 vs. Control group using unpaired t test. ## *P* < 0.01 and ### *P* < 0.001 vs. Mimic group. Mimic, stable overexpression of miR-150-5p. OE-FNDC5, table overexpression of FNDC5. Mimic + OE-FNDC5, co-transfection of plasmid overexpressing FNDC5 and plasmid overexpressing miR-150-5p

It is well known that the p38/MAPK pathway regulates the cell proliferation and osteogenic differentiation of BMSCs [19, 20], and it is an important downstream effector pathway for FNDC5 [21, 22]. Therefore, to determine whether miR-150-5p regulates osteogenic differentiation of BMSCs through the FNDC5/p38/MAPK signaling axis, we determined the expression of p38 and p-p38 protein using western blot. The result is shown in Fig. 5, the expression of p38 protein did not change due to overexpression of miR-150-5p and/or FNDC5. However, overexpression of miR-150-5p alone significantly decreased the expression of p-p38 and p-p38/p38 ratio, while FNDC5 overexpression alone strongly increased the expression of p-p38 and p-p38/p38 ratio. Importantly, the expression level of p-p38 and p-p38/p38 ratio in BMSCs co-overexpressed miR-150-5p and FNDC5 was significantly higher than that of miR-150-5p overexpressed alone.

### Irisin alleviates osteoporosis through the p38/MAPK pathway

**Fig. 6:**
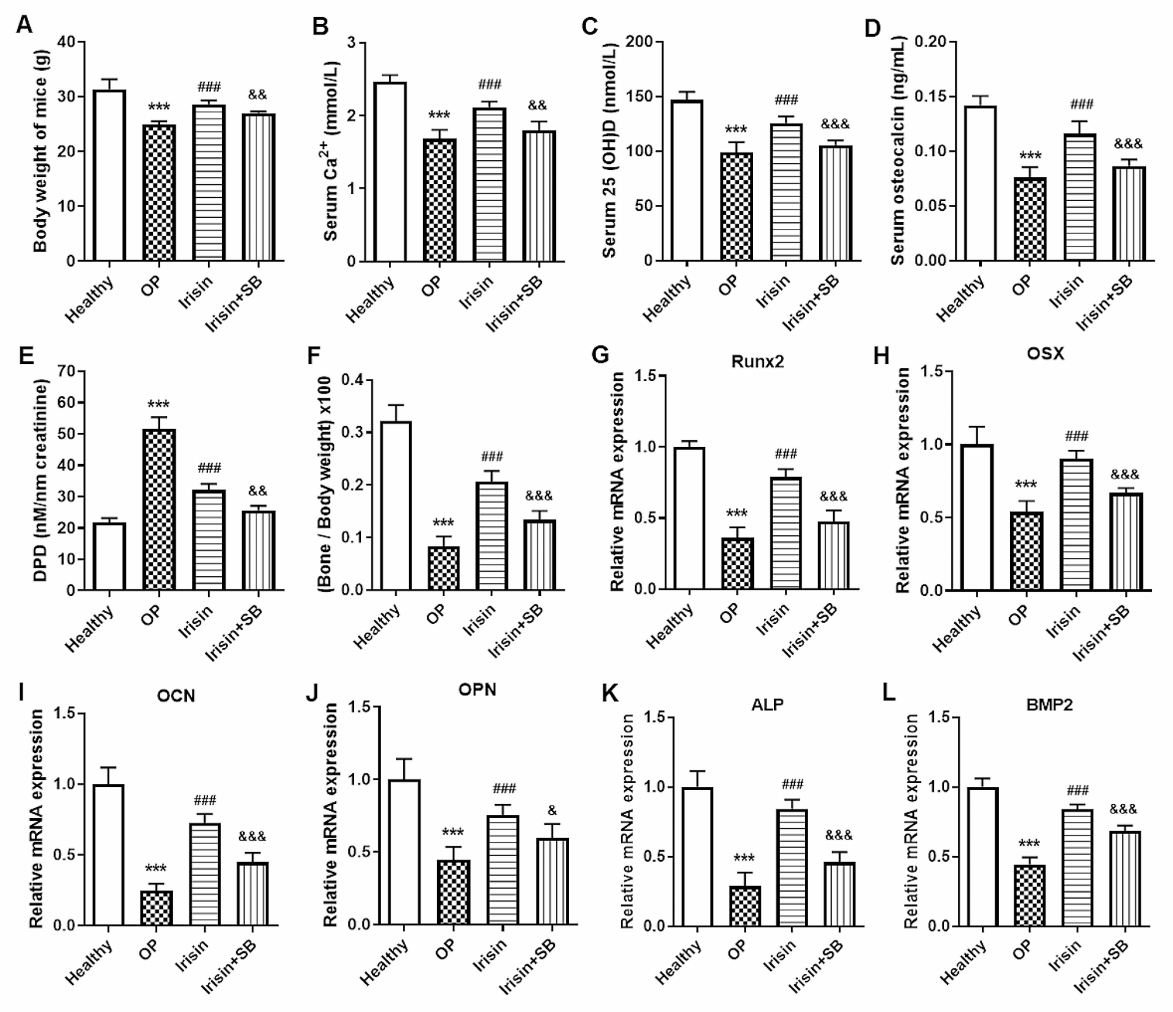
Irisin alleviates osteoporosis through the p38/MAPK pathway in vivo. (**A-F**) After completing the expected treatment with irisin and p38 inhibitors, we measured the level of the body weight (A), serum Ca^2+^ (B), serum 25 (OH) D (C), serum osteocalcin (D) and urinary DPD (E) in osteoporosis mice, and detected the weight of tibia and femur at 8th weeks (F). (**G-L**) After completing the expected treatment with irisin and p38 inhibitors, we isolated mouse BMSCs to detect the mRNA expression of Runx2 (G), OSX (H), OCN (I), OPN (J), ALP (K) and BMP2 (L) using RT-qPCR. 5 mice in each group. ***P < 0.001 compared with Healthy group; ^###^*P* < 0.001 compared with OP group; ^&^*P* < 0.05, ^&&^*P* < 0.01 and ^&&&^*P* < 0.001 compared with Irisin group. OP, osteoporosis mice. Irisin, osteoporosis mice treated with irisin. Irisin + SB, osteoporosis mice treated with irisin and p38 inhibitors (SB203580, SB)

Irisin, a peptide that is cleaved by FNDC5 and released into the bloodstream, is also a new hormone that promotes metabolic health [23]. The above results showed that miR-150-5p inhibited osteogenic differentiation of BMSCs by inhibiting FNDC5/Irisin expression. Therefore, increasing the level of FNDC5/Irisin in vivo can promote the osteogenic differentiation of BMSCs, which is beneficial for patients with osteoporosis. Herein, we established an osteoporosis mice model by subcutaneous injection of dexamethasone and treated osteoporosis mice with intraperitoneal injection of Irisin. The results showed that the body weight (Fig. 6A), serum Ca^2+^ (Fig. 6B), serum 25 (OH) D (Fig. 6C), serum osteocalcin (Fig. 6D) in the osteoporosis (OP) group were all lower than that in Healthy group and Irisin treatment (Irisin) group, but the level of DPN in urine in OP group was significantly higher in OP group than that in Healthy group and Irisin group (*P* < 0.001, Fig. 6E). At the same time, we measured the weight of tibia and femur at 8th weeks of Irisin treatment, and results showed that compared to Healthy group, the weight of tibia and femur / body weight in the OP group were significantly reduced, while the weight of tibia and femur / body weight in Irisin group were significantly higher than that in OP group (Fig. 6F). Importantly, SB203580 (SB), an inhibitor of the p38/MAPK pathway, significantly weakened the therapeutic effect of Irisin on osteoporosis in mice (Fig. 6A-F). At the same time, we also found that Irisin treatment significantly increased the expression of osteogenic genes (Runx2, OSX, OCN, OPN, ALP and BMP2) in BMSCs of osteoporosis mice, while SB203580 strongly weakened this function of Irisin (Fig. 6G-L).

## Discussion

Imbalance in bone formation and bone resorption is the direct cause of osteoporosis, and promoting bone formation and inhibiting bone resorption are the key to the prevention and treatment of osteoporosis [24, 25]. Bone cells originate from the differentiation of various stem cells, especially BMSCs. BMSCs are important members of the stem cell family, which are widely used in tissue regeneration, especially in cartilage damage and osteoporotic fractures because of their wide origin, easy isolation, strong proliferation and multi-directional differentiation potential [4, 5]. BMSCs currently lack uniform specific phenotypic markers, and most rely on their morphology, phenotype, and multidirectional differentiation capabilities to assist in identification [26, 27]. In this study, the isolated BMSCs were cultured and passed, and the cell morphology after passage was similar to the primary generation, mainly spindle-shaped, and the third-generation cells were positive for CD29 and CD90, while CD34 and CD45 were negative, so it could be confirmed that the isolated cells were BMSCs. Under certain induction conditions, BMSCs have the ability to differentiate into osteoblasts, adipocytes, chondrocytes, smooth muscle cells and other mesoderm cells, so it is an ideal seed cell in bone tissue engineering [28, 29].

In our study, we found that miR-150-5p not only inhibits the proliferation of BMSCs, but also inhibits the osteogenic differentiation of BMSCs, which is consistent with the results of Geng Y, et al. [13]. Geng Y, et al. found that the expression of miR-150-5p in BMSCs of senile osteoporosis patients was higher than that in BMSCs of healthy people, and was a hub gene of senile osteoporosis. Historically, non-coding RNAs have been considered “junk genes” due to they do not code for proteins, and they were not valued until they were discovered to indirectly regulate protein expression by regulating coding RNA expression. Currently, many miRNAs have been identified to regulate the osteogenic differentiation of BMSCs through target genes, such as miR-664a-5p was found to promote the osteogenic differentiation of BMSCs by directly decreasing HMGA2 expression [30], and miR-1271-5p inhibited the osteogenic differentiation of BMSCs by targeting FOXO1 [31]. Meaningfully, we found that FNDC5 was a target gene of miR-150-5p, which was further validated by luciferase gene reporter system, RT-qPCR, and western blotting. Importantly, overexpression of FNDC5 reversed the inhibition of osteogenic differentiation of BMSCs caused by upregulation of miR-150-5p, which suggested that miR-150-5p inhibited the osteogenic differentiation of BMSCs by inhibiting FNDC5 expression.

The protein encoded by the FNDC5 gene contains 209 amino acids, which are cleaved and translated to produce a 112-amino acid polypeptide fragment, i.e. irisin. Irisin is also known as the muscle factor, muscle and bone belong to the same motor system, not only anatomically proximity, but also closely coupled in terms of paracrine and endocrine signals in physiological and pathological situations, so irisin is thought to play an important role in bone metabolism [32]. Previous studies have shown that irisin acts directly on osteoblasts [33, 34]. Intraperitoneal injection of irisin results in increased trabecular and cortical bone thickness and an increase in the number of osteoblasts in subcutaneous WAT and simultaneously induces UCPl expression [33]. In vitro studies have shown that irisin increases osteoblast and mineralization, and inhibits nuclear factor-kB ligand (RANKL)-induced receptor activation in osteoclasts [34]. Importantly, FNDC5 / irisin has been found to play a therapeutic role in myocardial infarction [35] and cerebral infarction [17] by regulating the function of BMSCs.

Herein, we found that overexpression of FNDC5 promoted osteogenic differentiation of BMSCs in vitro, while intraperitoneal injection of irisin improves symptoms in osteoporosis mice. Furthermore, we also found that p38/MAPK pathway inhibitor not only weakened the function of FNDC5 to promote osteogenic differentiation in vitro, but also weakened the ameliorating effect of irisin on osteoporosis mice in vivo. Therefore, our results indicated that FNDC5 / irisin promoted the osteogenic differentiation of BMSCs by activating the p38/MAPK pathway, thereby improving the symptoms of osteoporosis mice. All the time, the activation of the p38/MAPK signaling pathway is thought to be related to the promotion of osteogenic differentiation of BMSCs, such as LL-37 [3], αCGRP [36] and Cannabidiol [37] have all been found to promote the osteogenic differentiation of BMSCs by activating the p38/MAPK pathway.

## Conclusion

In conclusion, miR-150-5p blocks p38/MAPK pathway conduction by inhibiting FNDC5 expression, thereby inhibiting osteogenic differentiation of BMSCs in vitro. In addition, intravenous administration of FNDC5 / irisin improves symptoms and promotes osteogenesis-related gene expression in osteoporosis mice by activating the p38/MAPK pathway.

### Electronic supplementary material

Below is the link to the electronic supplementary material.

Supplementary Material 1

## Data Availability

No datasets were generated or analysed during the current study.
