# Supplementary material for: mir-150-5p inhibits the osteogenic differentiation of bone marrow-derived mesenchymal stem cells by targeting irisin to regulate the p38/MAPK signaling pathway
Source: J Orthop Surg Res. 2024 Mar 18;19:190. doi: 10.1186/s13018-024-04671-6 (PMC10949585; doi:10.1186/s13018-024-04671-6)
Supplement: Supplementary file 1 — Supplementary Material 1 [file 13018_2024_4671_MOESM1_ESM.docx]

Table 1. Primers used in RT-qPCR analysis and FNDC5 sequence for luciferase reporter

| Gene name/ID | Primer sequences/ FNDC5 sequences |
| --- | --- |
| Alkaline phosphatase (ALP) | F: 5'-GTGGCCTCGACACAGAGAAA-3' |
|  | R: 5'-CAAAACCGCGTGGTTCACAT-3' |
| Runx2 | F: 5'-GCGGTGCAAACTTTCTCCAG-3' |
|  | R: 5'-TTCCTGCATGGACTGTGGTT-3' |
| Osterix (OSX) | F 5'-ACCGAGTCAGTGAGTGCTCTA-3' |
|  | R: 5'-GCTAACTCGCCTCCAGGTTT-3' |
| Osteocalcin (OCN) | F: 5'-TCACCGGTAAGTCTCGTCCT-3' |
|  | R: 5'-GCCTCAAGAGCTCCAGTTGT-3' |
| Osteopontin (OPN) | F: 5'-GCTCTTGAGGCAGCTTACCA-3' |
|  | R: 5'-AGGCCACCGTTGGATTTGAT-3' |
| Bone morphogenetic protein 2 (BMP2) | F: 5'-GAGTGCTCTAACCACAGTCCA-3' |
|  | R: 5'-GGGAGCTGCCGAGTCAATAA-3' |
| FNDC5 | F: 5'-CACTTCTTGTTGTGGGCTGC-3' |
|  | R: 5'- TGGGGTGGAGGGGATTAGAG-3' |
| β-actin | F: 5'-GGCTGTATTCCCCTCCATCG-3' |
|  | R: 5'-CCAGTTGGTAACAATGCCATGT-3' |
| FNDC5-WT | F: 5'-AAAUGCACAAAGCCCUGGGAGAU-3' |
| FNDC5-MUT | R: 5'-AAAUGCACAAGCCCACCCUCU-3' |
